# Supplementary material for: Association of maternal heavy metal exposure during pregnancy with isolated cleft lip and palate in offspring: Japan Environment and Children’s Study (JECS) cohort study
Source: PLoS One. 2022 Mar 24;17(3):e0265648. doi: 10.1371/journal.pone.0265648 (PMC8947080; doi:10.1371/journal.pone.0265648)
Supplement: S1 Table — (DOCX) [file pone.0265648.s001.docx]

**S1 Table. Major congenital anomalies recorded in Dr0m and Dr1m**^1^

| **External anomaly** | Anencephaly, Ablepharon, Microphthalmos/anophthalmos, Cataract, Facial cleft,  Omphalocele, Gastroschisis, Anal atresia, Cryptorchidism, Hypospadias, Polydactyly of hand, Syndactyly of hand, Split hand, Polydactyly of foot, Syndactyly of foot, Split foot, Upper limb defect, Lower limb defect, Myelomeningocele/spina bifida |
| --- | --- |
| **Internal anomaly** | Hydrocephalus, Holoprosencephaly, Diaphragmatic hernia, Esophageal atresia, Duodenal atresia, Jejuno-ileal atresia, Patent ductus arteriosus, Hypoplastic left heart syndrome, Pulmonary atresia, Tetralogy of Fallot, Transposition of the great artery, Tricuspid atresia, Truncus Arteriosus, Total anomalous pulmonary venous connection |
| **Chromosomal anomaly** | 21-trisomy, 18-trisomy, 13-trisomy |

1: Cleft lip, cleft palate, and cleft lip and palate are also available, but are not listed here for simplicity.
