# Supplementary material for: Association of maternal heavy metal exposure during pregnancy with isolated cleft lip and palate in offspring: Japan Environment and Children’s Study (JECS) cohort study
Source: PLoS One. 2022 Mar 24;17(3):e0265648. doi: 10.1371/journal.pone.0265648 (PMC8947080; doi:10.1371/journal.pone.0265648)
Supplement: S2 Table — (DOCX) [file pone.0265648.s002.docx]

**S2 Table: Heavy metal concentrations**^1^

| **Metal** | **This study** (N= 96,696) | **Previous study**^2^ (N=20,000) |
| --- | --- | --- |
| Mercury (μg/L) | 3.81 (2.67-4.21) | 3.83 (2.70-5.43) |
| Lead (μg/dL) | 0.61 (0.50-0.77) | 0.63 (0.51-0.78) |
| Cadmium (μg/L) | 0.70 (0.52-0.95) | 0.70 (0.52-0.95) |
| Manganese (μg/L) | 16.2 (13.2-19.7) | 16.1 (16.0-16.2) |
| ~~Selenium (μg/L)~~ | ~~177 (164-191)~~ | ~~178 (165-192)~~ |

1: Median with interquartile range

2: Adapted from Nakayama et al. *J Expo Sci Environ Epidemiol* 2019; 29: 633 (Ref. #18)
