# Supplementary material for: Association of maternal heavy metal exposure during pregnancy with isolated cleft lip and palate in offspring: Japan Environment and Children’s Study (JECS) cohort study
Source: PLoS One. 2022 Mar 24;17(3):e0265648. doi: 10.1371/journal.pone.0265648 (PMC8947080; doi:10.1371/journal.pone.0265648)
Supplement: S3 Table — (DOCX) [file pone.0265648.s003.docx]

**S3 Table. Multivariate analysis with log-scale metal concentration**

| **Heavy metals** | **Multivariate model**^1^(OR with 95% CI) | **p-value** |
| --- | --- | --- |
| log (Hg) | 1.11 (0.70-1.78) | 0.65 |
| log (Pb) | 1.03 (0.70-1.53) | 0.87 |
| log (Cd) | 0.90 (0.68-1.20) | 0.47 |
| log (Mn) | 0.93 (0.54-1.62) | 0.80 |

^1^Adjusted for sex and concentrations of the four metals.

OR: odds ratio, CI: confidence interval.
