# Supplementary material for: Association of maternal heavy metal exposure during pregnancy with isolated cleft lip and palate in offspring: Japan Environment and Children’s Study (JECS) cohort study
Source: PLoS One. 2022 Mar 24;17(3):e0265648. doi: 10.1371/journal.pone.0265648 (PMC8947080; doi:10.1371/journal.pone.0265648)
Supplement: S4 Table — (DOCX) [file pone.0265648.s004.docx]

**S4 Table. Detailed distribution data of heavy metal concentrations.**

| **Heavy metals**  (μg/L) | **Primary cohort**  (n=95,092) | **Isolated cleft L/P**  (n=192) | **Control**  (n=1920) |
| --- | --- | --- | --- |
| **Hg** | median^1^: 3.63 (2.54-5.19)  mean^2^: 4.20 (2.49)  range: 0.18-58.8 | median^1^: 3.64 (2.60-4.98)  mean^2^: 4.08 (2.38)  range: 0.78-21.7 | median^1^: 3.54 (2.52-5.27)  mean^2^: 4.29 (2.75)  range: 0.62-34.1 |
| **Pb** | median^1^: 5.85 (4.70-7.33)  mean^2^: 6.34 (2.86)  range: 1.20-110.0 | median^1^: 5.84 (4.49-7.18)  mean^2^: 6.22 (2.20)  range: 2.70-15.6 | median^1^: 5.75 (4.69-7.14)  mean^2^: 6.18 (2.31)  range: 1.22-25.9 |
| **Cd** | median^1^: 0.66 (0.50-0.90)  mean^2^: 0.75 (0.38)  range: 0.10-5.33 | median^1^: 0.66 (0.49-0.90)  mean^2^: 0.73 (0.33)  range: 0.24-2.12 | median^1^: 0.66 (0.49-0.89)  mean^2^: 0.73 (0.35)  range: 0.15-3.51 |
| **Mn** | median^1^: 15.4 (12.6-18.7)  mean^2^: 16.0 (4.67)  range: 3.1-60.8 | median^1^: 15.2 (12.5-18.5)  mean^2^: 16.0 (4.98)  range: 7.3-33.1 | median^1^: 15.4 (12.7-18.5)  mean^2^: 16.0 (4.78)  range: 5.8-41.8 |

1: with interquartile range

2: with standard deviation

Hg: mercury, Pb: lead, Cd: cadmium, Mn: manganese
