# Supplementary material for: Association of maternal heavy metal exposure during pregnancy with isolated cleft lip and palate in offspring: Japan Environment and Children’s Study (JECS) cohort study
Source: PLoS One. 2022 Mar 24;17(3):e0265648. doi: 10.1371/journal.pone.0265648 (PMC8947080; doi:10.1371/journal.pone.0265648)
Supplement: S5 Table — (DOCX) [file pone.0265648.s005.docx]

**S5 Table. Results from random-effects multivariate analysis (sensitivity analysis 2) ^1^**

| **Variable** | **OR (95% CI)** |
| --- | --- |
| **Age** (per 1 year increase) | 0.98 (0.95-1.01) |
| **FA intake (**per 1 unit increase**)** | 1.00 (1.00-1.00) |
| **Psychological stress (**Yes**)** | 1.12 (0.55-2.32) |
| **Alcohol**  Never  Ex  Current | (Reference)  0.82 (0.60-1.11)  0.59 (0.32-1.08) |
| **Smoking**  Never  Ex  Quit after pregnancy  Current | (Reference)  1.15 (0.81-1.63)  1.03 (0.67-1.62)  0.76 (0.34-1.71) |
| **Education**  High school  College  University or higher | (Reference)  0.79 (0.56-1.11)  0.97 (0.64-1.46) |
| **BMI (**per 1 unit increase**)** | 1.04 (1.00-1.08) |
| **Pre-pregnancy DM** | NA (did not converge)^2^ |
| **Supplement intake (**Yes**)** | 1.68 (1.13-2.50) |

1: Results of “NA” in the questionnaire were omitted.

2: Possibly due to the small numbers

OR: odds ratio, CI, confidence interval, FA: folic acid, BMI: body mass index, DM: diabetes mellitus, NA: not available.
