# Supplementary material for: Association of maternal heavy metal exposure during pregnancy with isolated cleft lip and palate in offspring: Japan Environment and Children’s Study (JECS) cohort study
Source: PLoS One. 2022 Mar 24;17(3):e0265648. doi: 10.1371/journal.pone.0265648 (PMC8947080; doi:10.1371/journal.pone.0265648)
Supplement: Attachment — Submitted filename: Rev2_ResponseLetter.docx [file pone.0265648.s007.docx]

**Reviewer #1**

**Comment 1-1**

Abstract needs to be revised, especially the part of results, results of conditional logistic regression which test the association between heavy metal exposure and isolated cleft L/P needs to be demonstrated.

**Response 1-1**

Following this suggestion, we have added the results of conditional logistic regression analysis to the Abstract (ln. # 56-58).

“*the adjusted odds ratios (per 1 μg/L increase) with 95% intervals were 0.96 (0.91-1.03), 1.01 (0.94-1.08), 1.00 (0.61-1.63) and 1.00 (0.97-1.03) for Hg, Pb, Cd and Mn, respectively*”

**Reviewer #2**

**Comment 2-1**

It is better to attach the revision indicating the changes made. Furthermore, the authors also should add the line and page number of the new texts to make the review easy.

**Response 2-1**

We apologize for the inconvenience in the previous version. In this revision, we indicated the changes with the line number.
